# Resting State of Dementia of the Alzheimer’s Type and Healthy Older Adults Using fNIRS

**DOI:** 10.3390/pathophysiology32020020

**Published:** 2025-05-02

**Authors:** In-sop Kim, Jaejin Hwang, Chorong Oh, Richard J. Morris

**Affiliations:** 1 School of Allied Health and Communicative Disorders, Northern Illinois University, DeKalb, IL 60115, USA; 2 Department of Industrial and Systems Engineering, Northern Illinois University, DeKalb, IL 60115, USA; jhwang3@niu.edu; 3 Department of Hearing, Speech and Language Sciences, Ohio University, Athens, OH 45701, USA; ohc@ohio.edu; 4 School of Communication Science and Disorders, Florida State University, Tallahassee, FL 32306, USA; Richard.Morris@cci.fsu.edu

**Keywords:** fNIRS, Alzheimer’s disease, dementia of the Alzheimer’s type, oxygenated hemoglobin (HbO), SVM, brain activation, resting state, biomarkers

## Abstract

Background/Objectives: This study explores variations in brain activity between individuals with dementia of the Alzheimer’s type (DAT) and healthy older adults during a resting state using functional near-infrared spectroscopy (fNIRS). Methods: FNIRS measured brain activity in ten AD patients and six healthy individuals. A device with 16 channels was placed on each participant’s forehead to measure oxygenation levels while they kept their eyes closed. The data were analyzed using a support vector machine (SVM) model. Results: The results indicated differences in oxygenated hemoglobin (HbO) levels between the two groups. Specifically, HbO levels were generally higher in the dementia group in the left hemisphere, with a sharp increase after 26 s. Conversely, HbO levels were consistently lower in the right hemisphere of the dementia group. The SVM analysis demonstrated high accuracy in differentiating between the AD and healthy groups based on HbO levels. Conclusions: The study indicates that differences in brain activity during resting state can potentially distinguish people with DAT from healthy individuals. We found relatively reduced hemoglobin activity in the prefrontal areas of those with DAT. Furthermore, the concentration changes in the HbO in the left lateral prefrontal and right medial brain regions emerged as the most informative in distinguishing individuals with DAT from healthy individuals. The results of the current study show that this method could improve current DAT diagnostic practices due to its efficiency.

## 1. Introduction

Dementia of the Alzheimer’s type (DAT), whose leading cause is Alzheimer’s disease (AD), is the most common form of dementia and stands as the most prevalent neurodegenerative cognitive disorder among older individuals. Over 6.9 million Americans and more than 55 million people worldwide are impacted according to the Alzheimer’s Association [1]. DAT gradually reveals cognitive functioning impairments, including memory issues, attention deficits, executive functioning deficits, and language impairments [2]. Diagnostic tools, such as CT, MRI, CSF analysis, and lumbar puncture, have been utilized for this population. However, these methods are expensive and invasive. The results of these tests also depend significantly on the patient’s physical health and age, decreasing the efficacy of the method [3,4]. Hence, a need exists to find a more affordable and non-invasive approach.

Functional near-infrared spectroscopy (fNIRS) is a non-invasive brain imaging technique that measures cognitive neural activation changes. fNIRS has been used to monitor brain activity by measuring the relative concentration of oxygenation and deoxygenation changes indicating oxygenated hemoglobin (HbO), deoxygenated hemoglobin (HbR), oxygenation (Oxy; HbO-HbR), and total hemoglobin (HbT). The fNIRS technology has advantages over fMRI, including its portability, ease of use, and insensitivity to motion artifacts, making it suitable for use with children, the elderly, and neurogenic populations and functional in a variety of settings [5,6,7]. FNIR provides a more accessible interaction and assessment of cognitive functions during cognitive tasks compared to other neuroimaging techniques.

Previous fNIRS studies have reported relatively lower HbO in the prefrontal regions in individuals with AD compared to healthy adults during cognitive tasks [8,9,10,11]. Specifically, Arai et al. (2006) found significantly lower HbO in the frontal and parietal lobes for individuals with AD, whereas the MCI group showed lower HbO activation only in the parietal lobe in the right hemisphere compared to the healthy control group [9]. In the MCI group, the HbO level was also found to be significantly reduced in the parietal region of the right hemisphere. Herrmann et al. (2008) also investigated the prefrontal cortex activity during a verbal fluency task in individuals with AD compared to healthy controls [10]. They demonstrated that the brain activation for the AD group was significantly decreased in the dorsolateral prefrontal cortex areas.

Yap et al. (2017) investigated brain activation in the prefrontal regions during semantic fluency tasks in individuals with mild AD, MCI, and healthy control groups [11]. They found that the AD group showed the lowest HbO levels, and the MCI group showed relatively higher HbO levels compared to the AD and the control group. However, the MCI group demonstrated a trend toward higher mean activation in both the right and left prefrontal cortex, followed by the healthy controls, with the mild AD group showing the lowest activation. In contrast, compensatory brain activity in the mild AD group declined. Additionally, in the MCI group, left prefrontal cortical oxygenation showed a moderately positive correlation with Mini-Mental State Examination scores, suggesting that greater neural “effort” (hyperactivation) may help sustain cognitive function. This correlation was not observed in healthy controls or individuals with mild AD. The authors concluded that higher brain activation in MCI represents a compensatory effort, suggesting that the brain requires more resources to complete the cognitive task. In contrast, the reduced brain activation in AD indicates an inability to effectively engage compensatory resources.

Cognitive function related to brain activation in DAT is significantly affected by the progressive decline, which leads to neural activation in the prefrontal cortex being commonly reported. However, the neural activities observed during the resting state remain unclear. A clear understanding of the brain activation patterns in this population is essential to distinguish dementia from healthy normal aging. Early identification of DAT is crucial for prompting intervention and opportunities for influencing the course of the disease. The purpose of this study is to investigate brain activation during a resting state in individuals with DAT compared to cognitively healthy elderly individuals.

## 2. Methods

### 2.1. Participants

This study was approved by the Institutional Review Board (approval number: HS23-0135). Two groups were included in this study: DAT and cognitively healthy. Interested people in the AD group were eligible to participate if they had a diagnosis of probable/possible DAT given by a board-certified neurologist or neuropsychologist and earned a score between 0 and 20 on the Saint Louis University Mental Status Exam (SLUMS). The inclusion criteria for the cognitively healthy group included no official diagnosis of cognitive impairment, no other neurological disorders, an earned score of 26 or higher on SLUMS. Following the consent and screening, 10 individuals with DAT (age M = 73, *SD* = 2.2; 4 males and 6 females) and 6 cognitively healthy adults (age M = 70, *SD* = 3.34; 1 male and 5 females) participated in this study. The participants were recruited from the communities in Chillicothe, OH, USA and Dekalb, IL, USA. The average SLUMS score for the DAT group was 15 (*SD* = 5.7) and 27.67 (*SD* = 1.11) for the control group.

### 2.2. Experiment

**Figure 1:**
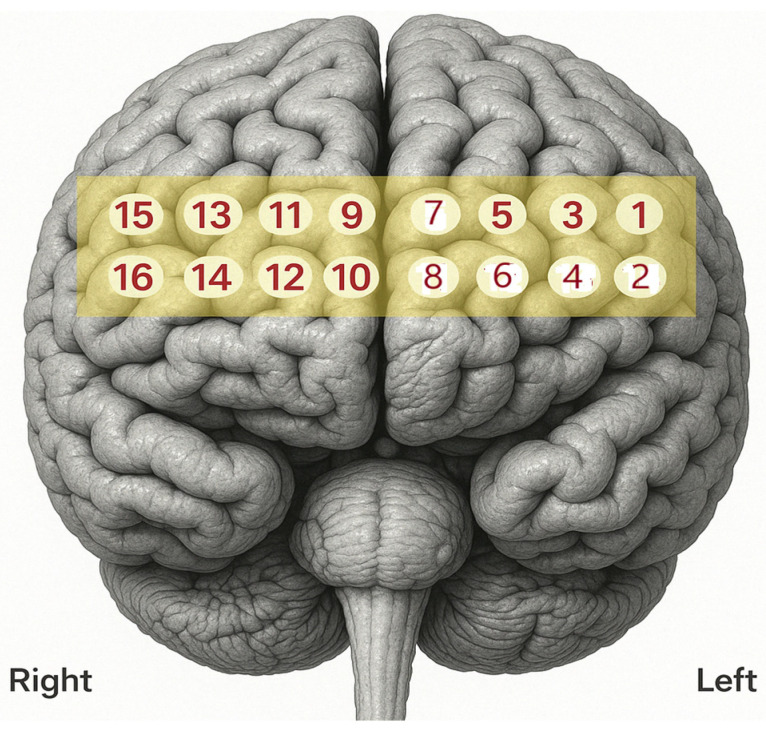
16 Channel optodes over prefrontal areas.

fNIRS was utilized to assess brain activity during a resting state. A 16-channel fNIRS band (fNIR 400 system, BIOPAC Systems, Inc., Santa Barbara, CA, USA) with four LED light sources and 10 detectors was fitted on all the participants’ foreheads (see Figure 1) over the international 10–20 electrode EEG placement F7, Fp1, Fp2, and F8 positions (sampling rate 2.0 Hz). Cognitive Optical Brain Imaging Studio and FNIRSOFT programs were used for data acquisition and analysis in conjunction with the fNIRS device. Markers were used on the Cognitive Optical Brain Imaging (COBI) software (version 1.4.0.9) to denote specific blocks. The fNIRS data were refined by using a sliding window motion artifact rejection algorithm to reduce the effects of motion interference on brain activation measurements. Moreover, a low-pass filter was applied to eliminate heart rate interference, and high-frequency noise was filtered out. To control lighting conditions during data collection, sessions were conducted in a laboratory with dimmed ambient light. Additionally, a black headband was placed over each participant’s forehead to block extraneous light from outside the lab. Prior to data collection, the Cognitive Optical Brain Imaging (COBI) software (version 1.4.0.9) was used to adjust and control the LED current to ensure optimal signal quality.

The relative concentrations of the average oxygenation values (µmol/L) in the prefrontal cortex were analyzed using the modified Beer–Lambert Law, while the participant closed their eyes for 40 s. HbO and HbT were used for the analysis during the resting state, as they were considered the most meaningful features in the data. The task took approximately 10 to 20 min to complete.

### 2.3. Support Vector Machine (SVM) Analysis

An SVM model was utilized with a linear kernel and regularization on the fNIR data rather than traditional statistical analysis. To capture the temporal dynamics present in the dataset, a sliding window approach was employed. A fixed window of 10 consecutive time steps was defined, and for each window, a series of summary statistics—including the mean, median, standard deviation, variance, skewness, and kurtosis—was calculated for every feature. The statistics were aggregated and flattened into a single vector per window, resulting in a new dataset with a multi-index structure that paired each summary statistic with its corresponding feature.

Following feature extraction, the data were split into training and testing sets using an 80/20 ratio. Preprocessing was integrated into a pipeline that first addressed missing values via imputation with the mean and then standardized the features using z-score normalization. This ensured that the data fed into the model were both complete and appropriately scaled. The performance of the SVM model was assessed on the testing set using various metrics, including the accuracy, confusion matrix, and classification report. To further understand the data distribution, violin plots were produced with seaborn to provide enhanced aesthetic insights into the density and spread of the data. In the current study, HbO was selected as the most meaningful index to classify individuals with DAT from cognitively healthy adults. Additionally, feature importance was computed to identify the most crucial brain regions for the classification task.

## 3. Results

### 3.1. fNIRS HbO Analysis

**Figure 2:**
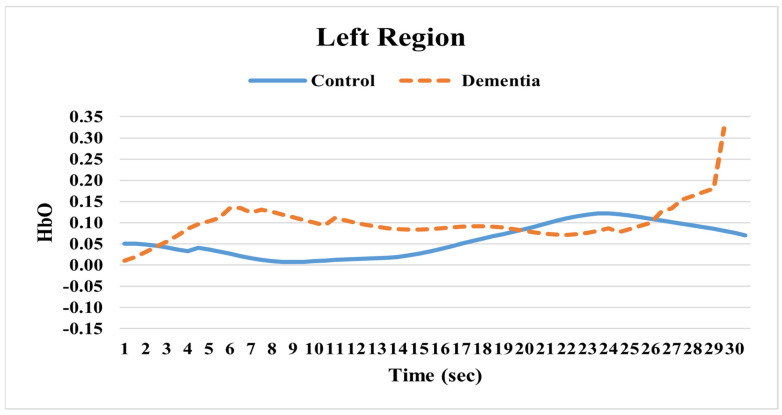
Oxygenated hemoglobin (HbO) for left hemisphere.

**Figure 3:**
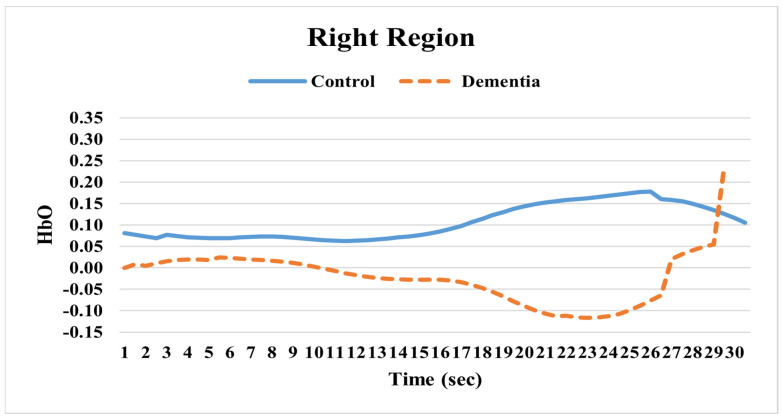
Oxygenated hemoglobin (HbO) for right hemisphere.

Examining the changes in HbO levels between individuals with AD and cognitively healthy adults over the measurement period revealed several notable differences. In the left hemisphere, the HbO levels in the dementia group were generally higher. After 26 s, a sudden increase in HbO levels for this group was observed (see Figure 2). In the right hemisphere, however, the dementia group’s HbO levels were consistently lower than those of the healthy group. Similar to the left hemisphere, there was also a sudden rise in HbO levels in the right hemisphere after 26 s (see Figure 3). Overall, the HbO activation in both the left and right hemispheres remained nearly identical between the two groups during the initial resting state period. However, around 20 s, the activity in both hemispheres of the dementia group significantly decreased. Then, at approximately 26 s, a sharp increase in activity was identified in the opposite direction (see Figure 2 and Figure 3).

### 3.2. SVM Performance

**Figure 4:**
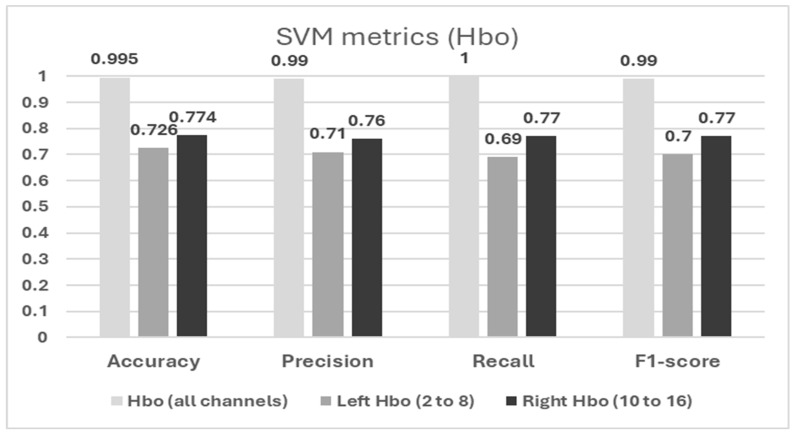
Support vector machine (SVM) metrics for oxygenated hemoglobin (HbO).

**Figure 5:**
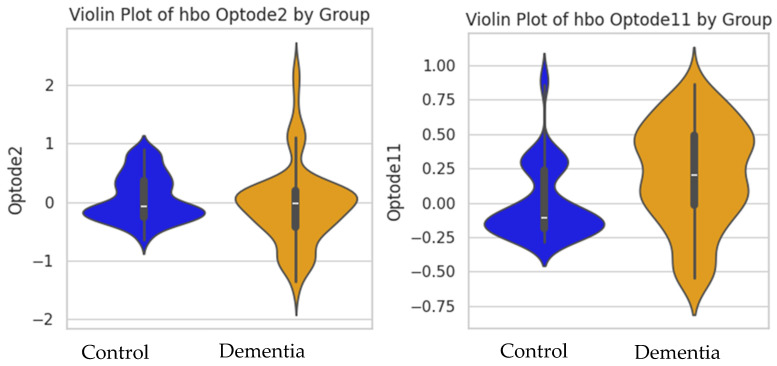
The violin plots of oxygenated hemoglobin (Hbo) for channels 2 and 11.

The SVM analysis revealed that considering all channels of the prefrontal cortex (optodes 1 to 16), HbO achieved exceptional performance with the highest recall (1.00), accuracy (0.995), precision (0.99), and F1-score (0.99) (see Figure 4). Furthermore, when evaluating the SVM model’s performance on either the left or right region, a decrease in performance was observed in individuals with DAT. Notably, the left regions’ HbO (optodes 2 to 8) showed the highest accuracy (0.726), precision (0.71), recall (0.69), and F1-score (0.7), with the median HbO value for optode 2 being the most contributing feature. In the right hemisphere (optodes 10 to 16), the results showed the highest accuracy (0.774), precision (0.76), recall (0.77), and F1-score (0.77), with the median HbO value for optode 11 being the most contributing feature (Figure 4 and Figure 5). The violin plots illustrate the distribution of the HbO across the DAT and control groups (see Figure 5), showing that the DAT group had a larger variation and distinct distribution of shapes of HbO compared to the control group, especially in optodes 2 and 11.

## 4. Discussion

The present study is among the few exploring the neural activities that are distinguished between individuals with DAT and those with healthy cognition. In particular, this study examined the brain activation during the resting state characterized by the brain’s natural activity in the absence of external stimuli. Analysis using an SVM model on fNIR data unveiled noteworthy variations in HbO levels in the prefrontal areas between the DAT and healthy cognition groups.

Our study found relatively lower hemoglobin activity in the prefrontal areas in individuals with DAT. In sum, the fNIRS HbO analyses demonstrated that the right hemisphere activity in individuals with DAT declines more markedly than in the left hemisphere compared to cognitively healthy individuals. Recent studies investigating cortical activation in individuals with AD during a resting state revealed similar results. Ferdinando et al. (2023) compared prefrontal cortical activity between AD and healthy control groups [12]. The results from the study showed reduced structure and lower brain activity in the AD group. The study concluded that this group had more irregular cortical activation compared to healthy controls. Keles et al. (2022) also found lower brain activation in both left and right prefrontal areas in the AD group [13]. In the current study, there was a significant HbO decrease at 25 s and a sharp HbO activation increase at approximately 26 s in the right hemisphere of the DAT group. Extending the resting state period beyond 25 s seems to trigger a sudden increase in activation. The reason for this change is unclear. This may suggest that right hemisphere brain activity is significantly lower than left hemisphere brain activity when compared to healthy individuals. This also suggests that overall right hemisphere activity is substantially reduced relative to that of healthy individuals as a distinct pattern. This lower brain activation level in the right hemisphere during a resting state may be related to the nature of this neurodegenerative disease. Therefore, this indicates that the DAT group has reduced HbO levels in the right prefrontal areas compared to the healthy elderly group during a resting state, regardless of whether cognitive tasks are involved. This phenomenon can be utilized as one of the rapid diagnostic methods to distinguish individuals with DAT from cognitively healthy individuals.

The SVM results showed that the model including both left and right hemisphere HbO (all channels) led to the highest performance of distinguishing between DAT and healthy cognition groups, with minimal errors. When considering data from left and right hemispheres individually, the right hemisphere’s HbO showed better performance than the left hemisphere. This was also supported by the violin plot showing that the DAT group showed a distinct data distribution shape and variations in HbO compared to the healthy cognition group. This was also aligned with our time series plot showing that the difference in HbO between groups was more apparent in the right hemisphere. Right prefrontal areas are related to executive functions, attention, and working memory—cognitive domains that are critically impacted in DAT. Furthermore, while aforementioned studies have reported poor left-hemisphere activation in individuals with Alzheimer’s disease during cognitive tasks, it remains unclear whether this pattern also emerges during the resting state. These findings underscore the significance of combining left prefrontal and right prefrontal feature sets, providing a more comprehensive representation of brain activity data and consequently leading to improved classification performance. Moreover, the concentration changes in the HbO in the left lateral prefrontal (optode 2) and right medial (optode 11) brain regions emerged as the most informative in distinguishing individuals with DAT from cognitively healthy individuals. Our results of the SVM analysis suggest that alterations in HbO in the right prefrontal cortex may serve as a reliable biomarker for detecting DAT. In addition, considering the Hbo data of both hemispheres improved the classification performance of the machine learning mode. In other words, while the right hemisphere showed the major contribution to the model performance, the left hemisphere still showed valuable input to improve the model’s overall performance. The results suggest the importance of multichannel neural analyses of various frontal regions to improve the early identification and screening of dementia.

There are some limitations in this study. First, the sample size was small, and the numbers in each group were not balanced. Second, a more in-depth examination of specific regions of interest within the prefrontal cortex may be required to accurately differentiate neural activation patterns between and healthy control groups. Third, although the SVM model showed promising results in this study, other types of machine learning models could be explored to see whether additional insights could be obtained from the data. For instance, ensemble learning, neural networks, and tree-based algorithms could capture the complex and non-linear relationship of the data. In addition, further parameter tuning and rigorous cross-validation methods would enhance the model’s performance and robustness to distinguish authentic neural patterns with dementia. Fourth, the on-and-off effects of medications taken by individuals with DAT on brain activation patterns should be considered to more accurately investigate the relative concentrations of oxygenated hemoglobin during resting states. Future investigations would benefit from a larger sample size and including broader brain regions in order to gain a more comprehensive analysis of both hemispheres during a resting state, since the existing assessment tool limits this scope. In conclusion, the findings in the current study suggest that resting-state neural activities diverge between the two groups, offering the potential for their distinct identification in cases. Such a method, which is both time- and cost-efficient, could enhance current practices in identification. Furthermore, by using fNIRS along with cognitive assessments, health professionals can have a more comprehensive interpretation of neurodegenerative diseases. Sometimes, traditional cognitive assessments may provide inconsistent results as they depend on the individual’s performance, which can vary based on their conditions such as fatigue or motivation. However, a combination of fNIRS and cognitive assessments can provide more valid and objective measurements, particularly during a resting state.

## Data Availability

The datasets presented in this article are not readily available because the data are part of an ongoing study and so are not publicly available.
